# Online dispute resolution for crypto-asset disputes: AI, smart contracts and digital inclusion by design

**DOI:** 10.3389/frai.2026.1929605

**Published:** 2026-10-09

**Authors:** Gioia Arnone, Marco Giacalone

**Affiliations:** Department of Private and Economic Law, Vrije Universiteit Brussel, Brussels, Belgium

**Keywords:** access to justice, artificial intelligence, digital inclusion, online dispute resolution, smart contracts

## Abstract

Crypto-asset disputes create distinctive barriers to effective remedies because they combine technical opacity, cross-border legal fragmentation, pseudonymous actors, irreversible transactions and uncertain enforcement pathways. These barriers are especially significant for vulnerable and non-expert users, who may be unable to identify the legally relevant harm, organize blockchain-based evidence, select an appropriate forum or obtain practical enforcement. This article develops a conceptual, normative and legal-design proposal for a hybrid Online Dispute Resolution (ODR) architecture that integrates off-chain artificial intelligence (AI) assistance with a minimized on-chain smart-contract layer. The model assigns AI systems a supporting role in legal information, triage, claim classification, evidence organization, explanation and negotiation, while reserving contested legal determinations for human review. A permissioned distributed ledger records only hashes, timestamps, procedural events, escrow states and execution proofs; personal data, communications, evidence and AI processing remain off-chain. Smart contracts are used as bounded enforcement tools for registration, escrow and execution of validated settlement terms, rather than as autonomous adjudicators. The article applies the architecture to three illustrative scenarios: asset seizure and recovery, exchange failure and mass claims, and crypto scams involving cross-border fraud. It then evaluates the model against European requirements concerning artificial intelligence, crypto-asset services, data protection, procedural fairness, cybersecurity and accountability. The contribution is twofold. First, it advances the concept of access to remedies by design, under which comprehension, participation, contestability, evidentiary integrity and enforceability are embedded into the architecture of digital dispute resolution. Second, it provides a technically specified evaluation framework for future prototyping and empirical validation. The article does not claim measured improvements in accessibility or efficiency; rather, it identifies testable design requirements and performance indicators for subsequent pilots, user studies and regulatory sandboxes.

## 1 Introduction

The rapid expansion of crypto-asset ecosystems has transformed the landscape of digital finance, enabling decentralized transactions, new investment models, and innovative forms of value exchange (Kayani and Hasan, 2024). At the same time, it has generated a growing range of disputes, including fraud and scam-related losses, unauthorized transactions, transfers to incorrect addresses, failures of crypto-asset service providers, disputes arising from decentralized finance protocols, and conflicts concerning smart-contract vulnerabilities or governance failures. Existing mechanisms for resolving these disputes remain fragmented and are often inaccessible to the users most affected (Ozili, 2022; Alamsyah et al., 2024). The resulting access-to-justice problem is not limited to formal access to courts. A user must first recognize the nature of the harm, identify the responsible actor, understand the available legal pathway, preserve and present technically complex evidence, participate meaningfully in the procedure and obtain an outcome that can be implemented in practice. Crypto-asset disputes complicate each of these stages because they frequently involve pseudonymous participants, cross-border infrastructures, irreversible transactions and legal uncertainty (Prajapati et al., 2024).

This produces a paradox. Crypto-assets are often associated with financial inclusion, yet their practical operation may create new forms of exclusion. A person may formally control a wallet or token while lacking the technical knowledge, legal expertise or financial resources needed to obtain redress. Traditional judicial processes may be too slow, costly or jurisdictionally fragmented, whereas platform-based remedies may lack independence, transparency or procedural safeguards. Artificial intelligence (AI) and blockchain technologies offer complementary, but limited, responses to this problem: AI can support user interaction, legal information, triage, evidence organization and negotiation. Blockchain and smart contracts can provide verifiable registration, escrow, auditability and execution. Neither technology should be treated as an autonomous solution; AI may reproduce bias, generate misleading outputs or obscure responsibility, while smart contracts may execute rigid or erroneous conditions without sufficient sensitivity to law or context (Poncibò et al., 2025).

This paper addresses this gap by proposing a hybrid Online Dispute Resolution (ODR) model that integrates AI-driven legal assistance with smart contract-based enforcement mechanisms. The central research question guiding this study is: how can AI and smart contracts be designed and combined to enhance digital inclusion and access to justice in crypto-asset dispute resolution, particularly for vulnerable and non-expert users? To answer this question, the paper adopts a conceptual and design-oriented approach, developing a technical–legal architecture that distinguishes between off-chain and on-chain processes. In this model, AI systems operate primarily in the off-chain layer, supporting user interaction, dispute classification, and legal guidance through explainable and user-friendly interfaces. Smart contracts, by contrast, operate in the on-chain layer, enabling secure dispute registration, verification, and automated enforcement of settlement outcomes.

The proposed framework emphasizes a user-centric design aimed at reducing informational and procedural barriers. By integrating explainable AI tools, guided workflows, and simplified interfaces, the model seeks to make complex legal and technical processes more accessible to non-expert users. At the same time, the use of smart contracts enhances trust, transparency, and efficiency by automating key aspects of dispute resolution and reducing reliance on costly intermediaries. However, the integration of these technologies also raises important legal and regulatory challenges, including issues related to algorithmic bias, accountability, and compliance with emerging frameworks such as the European Union’s regulatory approach to AI and crypto-assets. To ground the analysis in real-world contexts, the paper incorporates selected case studies involving crypto-asset seizures, exchange failures, and scam-related disputes. These examples illustrate both the limitations of existing mechanisms and the potential of hybrid AI–smart contract systems to provide more inclusive and effective solutions. Ultimately, the paper contributes to the literature on AI and law by advancing a technically informed and legally grounded model for inclusive digital justice in decentralized financial environments (Bhumichai et al., 2024). Existing digital dispute-resolution models can broadly be divided into three technological trajectories. First, conventional ODR platforms digitize negotiation, mediation, case management and communication, but generally remain centralized and depend upon external mechanisms for evidentiary verification and enforcement. Secondly, AI-supported LegalTech systems increasingly provide legal information, triage, document analysis, prediction or negotiation assistance, but their contribution is usually concentrated on cognitive or decisional support rather than on the execution of dispute outcomes. Thirdly, blockchain-based and decentralized dispute-resolution models employ smart contracts, tokenised incentives, decentralized juries or on-chain arbitration to increase transparency and automate execution. Such systems generally prioritize decentralization and cryptoeconomic enforcement rather than accessibility for non-expert and vulnerable users, and they can create tensions concerning data protection, procedural contestability and the legal status of automated decisions.

The novelty of the present architecture does not therefore reside in the independent use of AI, ODR or smart contracts. Each has previously been examined in digital dispute resolution. Its contribution lies in their functionally constrained integration around access to remedies by design. Five characteristics distinguish the model. First, AI is deliberately confined to assistive functions (information, triage, evidence organization, explanation and negotiation) and is prevented from autonomously determining contested legal rights. Secondly, blockchain use is minimized: a permissioned ledger records integrity proofs, procedural states and validated execution events, while personal data, evidence, communications and AI processing remain off-chain. Thirdly, smart contracts operate as bounded execution mechanisms rather than autonomous adjudicators. Fourthly, the architecture integrates procedural safeguards (human review, contestability, explanation, cooling-off periods and escalation) into the technical workflow rather than treating them as external legal constraints. Fifthly, digital inclusion is operationalized through measurable requirements concerning comprehension, accessibility, usability, fairness and enforceability. The contribution is therefore a legal-technical governance architecture, rather than merely a combination of existing technologies.

The remainder of the paper is structured as follows. Section 2 examines the nature of crypto-asset disputes and the sources of digital exclusion in this domain. Section 3 explores the role of AI in enhancing legal accessibility, while Section 4 analyses the potential and limitations of smart contracts in dispute resolution. Section 5 presents the proposed hybrid ODR architecture, and Section 6 applies the model to selected case studies. Section 7 discusses legal and regulatory implications, followed by an evaluation framework in Section 8. Section 9 concludes and poses some ideas on future researches.

## 2 Crypto-asset disputes and digital exclusion

The expansion of crypto-asset markets has been accompanied by a corresponding increase in disputes arising from their use. These disputes are heterogeneous in nature and reflect the technical, economic, and legal specificities of decentralized systems. Common categories include fraud and scam-related losses (Ponzi schemes, phishing, investment scams), unauthorized transactions resulting from compromised private keys, errors in transaction execution (including irreversible transfers to incorrect addresses), disputes linked to the failure or insolvency of crypto-asset service providers (such as exchanges), and conflicts emerging from decentralized finance (DeFi) protocols, including smart contract vulnerabilities and governance failures. Unlike traditional financial disputes, these cases frequently involve pseudonymous actors, immutable transaction records, and infrastructures that operate beyond clearly defined jurisdictions (Wang et al., 2024; Dam et al., 2023).

These characteristics generate a complex dispute environment in which traditional legal frameworks and enforcement mechanisms are often inadequate. Jurisdictional uncertainty is a primary challenge: determining the applicable law and competent authority is difficult when transactions occur across borders and without identifiable intermediaries. Moreover, the evidentiary dimension of crypto disputes introduces additional complexity. While blockchain ledgers provide transparent and tamper-resistant records, interpreting transaction data requires a degree of technical expertise that is not widely accessible. The absence of standardized procedures for presenting and validating blockchain-based evidence further complicates judicial and extrajudicial resolution processes. Within this context, the notion of digital exclusion becomes central. Digital exclusion in crypto-asset disputes does not merely refer to a lack of access to digital tools but encompasses a broader set of barriers that prevent individuals from effectively understanding, navigating, and enforcing their rights.

Digital exclusion in crypto-asset disputes extends beyond the absence of internet access. It includes barriers that prevent individuals from understanding, navigating and enforcing their rights. These barriers can be grouped into three mutually reinforcing dimensions (Alessa, 2022):

**Table 1:** 

Dimension	Principal barrier	Effect on the user	Design response
Technical exclusion	Complex wallets, keys, addresses, transaction histories and irreversible operations.	Difficulty identifying the event, preserving evidence or recovering from error.	Guided evidence collection, plain-language explanations and technical validation.
Legal exclusion	Fragmented regulation, uncertain jurisdiction and unclear allocation of responsibility.	Difficulty identifying rights, forum, applicable law and available remedies.	Legal triage, jurisdictional prompts, escalation and institutional referral.
Cognitive exclusion	Specialized terminology and opaque legal or algorithmic reasoning.	Reduced comprehension, confidence and meaningful participation.	User-relative explanations, structured workflows and human assistance.

Technical exclusion arises because interaction with crypto-assets requires users to manage private keys, addresses, wallets, transaction hashes and security practices that are often unintuitive and unforgiving of error (Wronka, 2024). Legal exclusion results from the fragmented and evolving regulation of assets, intermediaries and remedies (Burri and Kugler, 2024). Cognitive exclusion concerns the user’s ability to understand both the technological event and its legal significance, even where information formally exists (Pocher, 2025).

The three dimensions reinforce one another. Technical opacity makes it difficult to identify legally relevant facts; legal fragmentation makes it difficult to convert those facts into a claim; and cognitive barriers prevent users from understanding the available route. The result may be false digital empowerment: formal control over digital assets without practical capacity to obtain redress.

Traditional courts remain indispensable but may be poorly adapted to low-value, technically complex and cross-border disputes. Private platform procedures can offer speed but may lack independence, transparency, contestability or enforceability (Babaie, 2024; Chan et al., 2023). The objective is therefore not to replace courts, regulators or human professionals, but to design an intermediate procedural infrastructure capable of improving comprehension, evidence preservation, coordination and settlement while preserving escalation to formal institutions (Kamalova, 2024). In this respect, digital inclusion should be understood as the practical capacity to enter, comprehend and use a dispute-resolution process, rather than as mere technical access to a platform (Rothe et al., 2025; Van Rothe et al., 2023). In crypto-asset disputes, inclusion requires that the user can describe the loss in ordinary language, receive assistance in identifying the relevant transaction, understand the possible legal characterization, preserve evidence, communicate with the other party, challenge errors and obtain an enforceable outcome.

This article refers to that objective as access to remedies by design. The concept links interface accessibility with procedural fairness and enforcement. It requires a system architecture in which explanations, user choice, evidence integrity, human review and execution safeguards are embedded from the outset rather than added after deployment.

Against this background, the integration of AI and blockchain technologies offers a promising avenue for addressing the challenges identified above (Kuznetsov et al., 2024; Rane et al., 2023). AI systems have the potential to reduce cognitive and procedural barriers (Marocco et al., 2024) by translating complex legal and technical information into accessible formats, guiding users through dispute processes, and supporting decision-making (Witt et al., 2024). At the same time, blockchain-based smart contracts can enhance trust and enforceability by automating key aspects of dispute resolution and providing transparent, verifiable records of interactions and outcomes. However, the realization of this potential depends on the careful design of hybrid systems that balance automation with human oversight and that prioritize inclusivity as a core design principle.

The following sections build on this analysis by examining, first, the role of AI in enhancing legal accessibility, and, second, the potential and limitations of smart contracts as tools for dispute resolution in crypto-asset ecosystems.

## 3 AI for legal inclusion

Artificial intelligence (AI) has emerged as a central component of contemporary Legal-Tech, offering new possibilities to enhance access to justice by supporting legal information, decision-making, and dispute resolution processes. In the context of crypto-asset disputes (Arnone and Giacalone, 2025), characterized by technical complexity, legal uncertainty, and cross-border dynamics, AI systems can play a particularly important role in reducing barriers to participation for vulnerable and non-expert users. However, the contribution of AI to digital inclusion (Santiváñez et al., 2025) depends not only on its functional capabilities, but also on its design, transparency, and governance. This section develops a functional taxonomy of AI tools for legal inclusion and examines key challenges related to explainability, bias, and human oversight.

### 3.1 A functional taxonomy of AI tools in crypto-dispute resolution

AI applications in legal contexts can be categorized according to the specific functions they perform within the dispute resolution process. In the domain of crypto-asset disputes, four core functional categories can be identified.

First, legal information and triage systems provide users with initial guidance on their legal situation. These systems, often implemented as conversational agents or chatbots (Arnone, 2024), use natural language processing (NLP) to interpret user queries and deliver tailored information in accessible language. In the context of crypto disputes, such tools can assist users in identifying the nature of their claim (fraud, unauthorized transaction, platform failure), understanding available remedies, and determining whether formal dispute resolution is appropriate (Zhuk, 2023; Frolova and Kupchina, 2023; Barbereau and Bodó, 2023). By lowering the threshold for engagement, triage systems address both informational and cognitive barriers.

Second, decision-support and classification systems assist in structuring and evaluating disputes. These tools can classify cases based on predefined categories, suggest relevant legal frameworks, and support the organization of evidence. In crypto-asset contexts, AI systems may help interpret transaction histories, flag suspicious patterns, or map user claims onto legal doctrines. While such systems do not replace human judgment, they can significantly enhance the efficiency and consistency of dispute handling (Ikeda et al., 2025; Carneiro et al., 2014).

Third, negotiation and mediation support systems facilitate interaction between parties during the dispute resolution process (Zeleznikow, 2021). AI can support negotiation by proposing settlement options, identifying areas of agreement, and modeling potential outcomes. In hybrid ODR environments, these tools can complement human mediators by providing data-driven insights while preserving the flexibility and contextual sensitivity of human intervention (Hussein, 2025). For vulnerable users, such support can mitigate asymmetries in knowledge and bargaining power (Casarosa, 2024).

Fourth, fraud detection and risk assessment systems play a preventive and diagnostic role. By analyzing large datasets and transaction patterns, AI can identify indicators of scams, money laundering, or other illicit activities. In dispute resolution contexts, these systems can support the validation of claims and the identification of responsible actors (Abedi et al., 2025). For users who lack technical expertise, automated risk assessment tools can provide critical support in understanding the nature and credibility of their claims.

Together, these functional categories illustrate the potential of AI to operate across the entire lifecycle of a dispute, from initial engagement to resolution (Hussein, 2025). Importantly, their effectiveness in promoting digital inclusion depends on their integration into user-centric systems that prioritize accessibility and comprehension.

### 3.2 Explainability and epistemic accessibility

A central condition for the use of AI in access to justice is explainability: the ability of a system to provide understandable and meaningful reasons for its outputs. In the context of crypto-asset disputes, where both legal and technical dimensions are complex, explainability is closely linked to epistemic accessibility, understood as the capacity of users to comprehend the information and processes that affect their rights (Pasquale, 2025; Borgesano et al., 2025).

Black-box AI systems, which produce outputs without transparent reasoning, risk reinforcing existing inequalities by privileging users with higher levels of technical or legal literacy.

For vulnerable and non-expert users, opaque decision-making processes may undermine trust and limit the practical usability of AI tools (Romeo and Conti, 2026; Wright et al., 2026). Accordingly, AI systems designed for legal inclusion should incorporate explainability mechanisms such as plain-language explanations of legal concepts and outcomes; step-by-step guidance through procedural stages; visual or interactive representations of decision logic; disclosure of system limitations and uncertainty. Explainability, in this sense, is not merely a technical feature but a normative requirement, closely tied to principles of procedural fairness and accountability (Buttaboni and Floridi, 2026). It enables users to understand, question, and effectively engage with AI-supported processes, thereby enhancing their agency within digital justice systems (Yadav and Ansari, 2025).

### 3.3 Bias, fairness, and risks of exclusion

While AI has the potential to reduce barriers to justice, it may also introduce or amplify forms of bias and exclusion (Panarese et al., 2026). Algorithmic bias can arise from multiple sources, including biased training data, flawed model design, or inappropriate application contexts (Hu et al., 2026). In the domain of crypto-asset disputes, where empirical data may be limited or unevenly distributed, the risk of bias is particularly significant.

Addressing these risks requires a proactive approach to fairness and accountability in AI design. Key strategies include ensuring diversity and representativeness in training data; conducting regular audits of system performance; implementing fairness constraints and bias mitigation techniques; providing mechanisms for user feedback and contestation (Bahangulu and Owusu-Berko, 2025).

From a legal perspective, these issues are increasingly addressed within emerging regulatory frameworks, which emphasize transparency, risk management, and human oversight in high-risk AI applications. Ensuring that AI systems for dispute resolution comply with such standards is essential to prevent the reproduction of exclusion within digital justice environments (Onțanu and Tjong Tjin Tai, 2025; Marra, 2025).

### 3.4 Human-in-the-loop and hybrid governance

A critical dimension of AI for legal inclusion is the integration of human-in-the-loop mechanisms, which ensure that human judgment remains central to decision-making processes (Lazaros et al., 2026; Saxena, 2026). In the context of dispute resolution, fully automated systems may lack the contextual sensitivity, ethical reasoning, and flexibility required to address complex or contested cases. This is particularly relevant for vulnerable users, whose circumstances may not be adequately captured by standardized models.

Hybrid systems that combine AI capabilities with human oversight offer a more balanced approach. In such systems, AI supports routine tasks, information processing, and preliminary assessments; human actors (e.g., mediators, legal advisors) retain authority over critical decisions; users are provided with opportunities to interact with both automated and human agents.

This model enhances both efficiency and fairness, enabling scalable solutions while preserving the safeguards associated with human judgment. Moreover, it aligns with emerging regulatory approaches that emphasize the importance of human oversight in high-impact AI applications.

### 3.5 Usability and user-centric design for vulnerable users

The contribution of AI to digital inclusion depends fundamentally on usability and user-centric design (Amato et al., 2026). AI systems that are technically sophisticated but difficult to use will fail to achieve their intended purpose. For vulnerable and non-expert users, usability considerations are particularly critical. Usability is a substantive condition of inclusion. The system should minimize jargon, use step-by-step workflows, support multiple languages and devices, permit users to pause and return, provide accessibility features and make human assistance visible at every critical stage. The interface should also avoid dark patterns. Users should not be pressured into settlement, required to waive rights without clear explanation, or directed toward automated execution by default. Consent should be granular, recorded and capable of withdrawal before execution becomes irreversible. In the context of crypto-asset disputes, these principles are essential to bridge the gap between complex technological infrastructures and the practical needs of users. AI, when designed with these considerations in mind, can act as a mediator between technical systems and human understanding, transforming complexity into actionable knowledge.

AI offers significant potential to enhance digital inclusion in crypto-asset dispute resolution by reducing informational, procedural, and cognitive barriers (Molinari and Giacalone, 2026). This potential is contingent upon the careful design of systems that are explainable, fair, and centered on human needs. The next section builds on this analysis by examining the role of smart contracts in enabling automated and trustworthy enforcement within hybrid dispute resolution frameworks.

## 4 Smart contracts as bounded verification and enforcement tools

Smart contracts constitute a core technological component of blockchain-based ecosystems and play an increasingly relevant role in the design of digital dispute resolution mechanisms. Defined as self-executing code deployed on a distributed ledger, smart contracts enable the automatic performance of predefined actions once specified conditions are met (Alevizos, 2025). In the context of crypto-asset disputes, they offer the potential to enhance enforcement, transparency, and efficiency by reducing reliance on intermediaries and minimizing transaction costs. However, their integration into dispute resolution processes raises important legal, technical, and governance challenges, particularly when considered through the lens of digital inclusion.

First, a smart contract may register the existence of a dispute by recording a hash and timestamp without publishing the underlying claim or personal data (Arsat et al., 2025). Second, it may hold controllable assets in escrow while the parties negotiate or a decision is reviewed (Ayodele et al., 2025; Kadioglu Kumtepe, 2025). Third, it may execute settlement terms after the parties and, where required, a human reviewer have validated those terms. Fourth, it may create an auditable record of procedural events and execution (Alhasan, 2026; Hochgürtel, 2026). These functions address the gap between decision and implementation. A settlement that depends on later voluntary performance may be ineffective where the parties are pseudonymous or located across jurisdictions. Conditional execution can improve reliability where the relevant assets are technically controllable and the legal conditions for execution have been satisfied.

From the perspective of digital inclusion, smart contracts offer several advantages. By automating enforcement processes, they reduce the need for costly legal representation and complex procedural steps (Giacalone and Arnone, 2025), thereby lowering barriers to entry for users with limited resources. The use of standardized and programmable mechanisms can also enhance predictability, allowing users to better understand the consequences of different dispute resolution paths. Moreover, smart contracts can support trust in low-trust environments.

In decentralized systems where traditional intermediaries are absent, users may be reluctant to engage in dispute resolution processes due to concerns about fairness or enforceability. Automated execution reduces reliance on third parties and can provide assurance that outcomes will be implemented as agreed.

In combination with AI-driven interfaces, smart contracts can also contribute to procedural simplification. While the underlying code may be complex, user-facing systems can abstract this complexity, presenting clear and actionable steps to users. In this way, smart contracts can operate as a backend enforcement layer that supports accessible front-end interactions.

Despite these advantages, the use of smart contracts in dispute resolution is subject to significant limitations.

One of the primary challenges is rigidity. Smart contracts execute predefined instructions and lack the flexibility to adapt to unforeseen circumstances or interpret ambiguous situations. In disputes that require contextual assessment or equitable considerations, purely automated execution may lead to outcomes that are technically correct but substantively unjust.

A second issue concerns legal validity and enforceability. While smart contracts can perform actions on a blockchain, their legal status varies across jurisdictions. Questions arise as to whether smart contract code constitutes a legally binding agreement, how errors or bugs should be treated, and how disputes involving smart contracts themselves should be resolved. In the absence of harmonized legal frameworks, these uncertainties may limit the adoption of smart contract-based dispute resolution mechanisms.

Third, security and technical vulnerabilities present significant risks. Smart contracts are susceptible to coding errors, exploits, and unforeseen interactions with other contracts. High-profile incidents in decentralized finance have demonstrated that vulnerabilities can lead to substantial financial losses. For vulnerable users, who may lack the expertise to assess such risks, these issues can exacerbate rather than mitigate exclusion.

Fourth, there are concerns related to accountability and liability. When a smart contract executes an outcome that is harmful or incorrect, identifying the responsible party (whether developer, platform, or user) can be challenging. This raises complex questions regarding the allocation of liability and the availability of remedies.

Given these limitations, the integration of smart contracts into dispute resolution systems requires a hybrid governance approach that combines automated execution with human oversight. Rather than replacing traditional legal processes, smart contracts should be understood as complementary tools that can enhance specific functions within a broader framework.

In a hybrid model, smart contracts are used primarily for registration and verification of disputes; management of escrow and conditional assets; execution of clearly defined settlement outcomes.

At the same time, human actors (such as mediators, arbitrators, or legal advisors) retain control over interpretative and discretionary aspects of dispute resolution. This division of roles allows the system to benefit from the efficiency and reliability of automation while preserving the flexibility and fairness associated with human judgment.

Importantly, hybrid governance also facilitates regulatory compliance. By maintaining off-chain processes for sensitive data and decision-making, systems can better align with legal requirements related to data protection, consumer protection, and procedural fairness. Smart contracts, in this context, operate within a controlled environment that mitigates risks while preserving their functional advantages.

For smart contracts to contribute effectively to digital inclusion, their design must be integrated into user-centric ODR systems. This entails abstracting technical complexity through intuitive interfaces; ensuring that users understand the implications of automated execution; providing safeguards against errors, including the possibility of human intervention or override mechanisms; embedding transparency and auditability in a way that is accessible to non-expert users.

In practice, this means that smart contracts should not be directly exposed to users as complex technical artefacts, but rather embedded within systems that translate their functionality into clear and manageable actions. When combined with AI-driven assistance, smart contracts can form part of a cohesive architecture that supports both accessibility and enforceability.

In conclusion, smart contracts offer powerful tools for enhancing the effectiveness and efficiency of dispute resolution in crypto-asset environments. However, their contribution to digital inclusion depends on their integration within hybrid systems that balance automation with human oversight and that prioritize usability and legal robustness. The next section builds on this analysis by presenting a hybrid ODR architecture that combines AI and smart contracts into a unified framework for inclusive digital justice.

## 5 A hybrid ODR architecture for digital inclusion in crypto-asset disputes

The architecture, not derived from technological convenience alone, follows a problem/requirement design logic. The three forms of exclusion identified in Section 2 (technical, legal and cognitive) are first translated into functional requirements. Technical exclusion generates requirements for guided evidence capture, abstraction of blockchain complexity and assisted verification. Cognitive exclusion requires plain-language interaction, explainability and user-confirmable AI outputs. Legal exclusion requires human review, contestability, jurisdictional escalation and separation between technological execution and legal determination. These requirements are then tested against three normative bodies of principle. First, access-to-justice theory requires not merely formal availability of a procedure but the practical ability to understand, enter, participate in and obtain an effective remedy through that procedure. Secondly, procedural-fairness principles require notice, participation, impartial review, reason-giving, contestability and meaningful human intervention where legally relevant judgments are made. Thirdly, human-centered system design requires that technological complexity be adapted to user capabilities rather than transferring the cognitive burden of the system to the user.

The architecture therefore employs a form of design triangulation: a design element is retained only where it responds to an identified user barrier, performs a necessary technical or procedural function and can be reconciled with applicable legal safeguards. For example, the separation between off-chain and on-chain processing is justified simultaneously by functional flexibility, data minimisation and the need to avoid immutable storage of personal evidence. Human review is not added merely as a precaution but follows from the risk that AI-generated classifications and recommendations may affect legal choices without adequately capturing context. Similarly, permissioned DLT is preferred to unrestricted public blockchain because the system requires identifiable governance, controlled validator participation, auditability and proportionate disclosure rather than maximum decentralization.

The design principles should therefore be understood as normatively derived design hypotheses. They are theoretically justified in the present conceptual study but remain empirically falsifiable: subsequent prototyping and user testing must determine whether the resulting architecture actually improves comprehension, participation, fairness and enforceability.

The reference architecture does not depend on a single proprietary model. Natural-language functions may be implemented through a large language model or smaller task-specific NLP models, whereas classification and fraud-risk functions may use supervised or rules-enhanced models appropriate to the relevant dataset. Retrieval-augmented generation should be preferred for legal information so that user-facing explanations are grounded in an approved, version-controlled corpus of legislation, guidance and procedural information. Model outputs should contain confidence or uncertainty information where technically meaningful. The AI layer must not autonomously determine liability, entitlement or enforceability.

Services should communicate through authenticated REST or equivalent APIs using structured schemas. Separate endpoints should govern identity, claim submission, evidence metadata, AI requests, mediation events, settlement validation and blockchain transactions. API gateways should provide authentication, authorisation, rate limiting, logging, schema validation and version control.

Authentication should follow a tiered model. Browsing and general information can remain anonymous. Filing a claim requires verified account identity; execution of settlements or transfer of controlled assets requires stronger authentication. eIDAS-compatible electronic identification and the European Digital Identity Wallet may constitute one implementation pathway, but alternative mechanisms must remain available to avoid identity-based digital exclusion.

User input enters the off-chain interface; identity data and evidence are stored in logically separated encrypted repositories; AI receives only the fields necessary for the relevant task; the case-management system records procedural decisions; only an authorized and validated procedural event can be transmitted through the blockchain connector; the ledger stores the resulting hash, timestamp, state change or execution proof. The blockchain layer never receives the full pleadings, communications or AI prompts.

A permissioned network is selected because the ODR environment requires identifiable governance and controlled access. The number and institutional independence of validators would be fixed during prototyping. PBFT-type consensus is preferable where Byzantine fault tolerance is required; Raft-type consensus may be sufficient where the governance model assumes non-malicious authenticated nodes. The final selection should be benchmarked for throughput, latency, fault tolerance and energy use (Figure 1).

**Figure 1:**
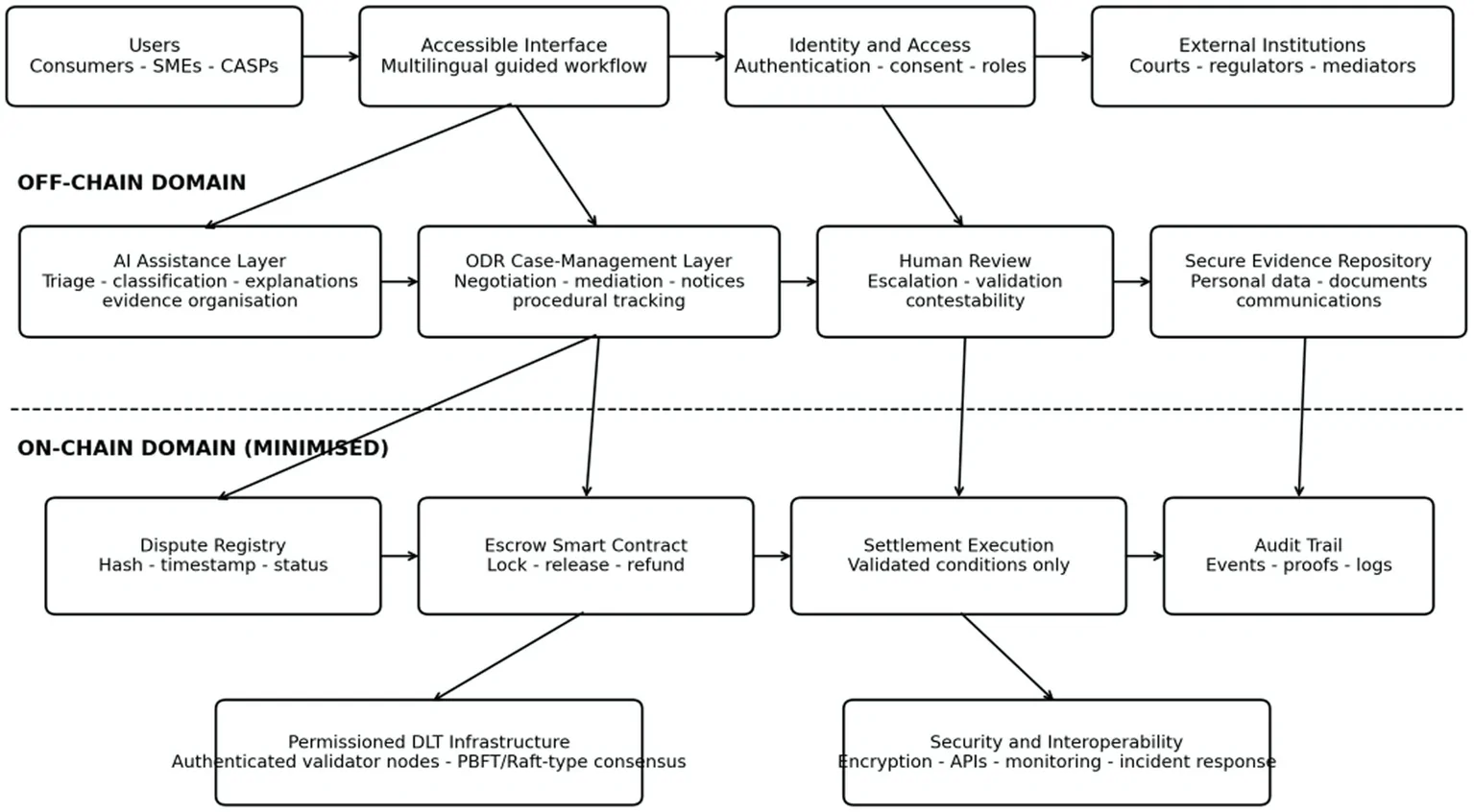
Technical and procedural architecture of the proposed hybrid ODR model.

The proposed system adopts a four-layer architecture, each performing distinct but interrelated functions:

1. User Interface Layer (Access and Interaction): This layer constitutes the entry point for users and is designed to maximize usability and accessibility. Key features include natural language interaction through conversational interfaces; step-by-step guided workflows; multilingual and inclusive design; compatibility with low digital literacy levels. Its primary function is to translate complex dispute processes into intuitive user experiences.
2. AI Assistance Layer (Off-Chain Cognitive Support): This layer provides the core intelligence of the system and operates off-chain. It includes legal triage systems to identify dispute types; classification engines to structure claims; explainability modules to simplify legal reasoning; fraud detection tools to assess risk and credibility. By reducing informational asymmetries and supporting decision-making, this layer directly addresses cognitive and procedural exclusion.
3. ODR and Mediation Layer (Hybrid Decision Space): This layer manages the dispute resolution process through a combination of automated and human-driven mechanisms case management and evidence handling; AI-assisted negotiation support; optional intervention by human mediators or legal experts. The integration of human-in-the-loop mechanisms ensures that complex or sensitive cases benefit from contextual evaluation, preserving procedural fairness.
4. Smart Contract and Blockchain Layer (On-Chain Enforcement): This layer operationalizes enforcement and trust through blockchain technology: dispute registration via cryptographic timestamping; escrow mechanisms for disputed assets; encoding of settlement terms into smart contracts; automated execution of outcomes; immutable audit trail of the process. By ensuring reliable enforcement without intermediaries, this layer addresses the enforcement gap typical of cross-border crypto disputes.

In other words, the user-interface layer provides multilingual, accessible and guided interaction. The AI assistance layer supports legal information, triage, classification, explanations and evidence organization. The ODR and mediation layer manages notices, communication, negotiation, human review and procedural status. The blockchain and smart-contract layer provides integrity proofs, escrow, validated execution and auditability. Identity and access management cuts across the layers. Users, mediators, platform personnel, CASPs and validators should be assigned role-based permissions. Authentication should be proportionate to the procedural act: initial information may be available with minimal identification, while claim submission, settlement and execution require stronger verification. The architecture also connects to external institutions. Courts, regulators, insolvency administrators, law-enforcement authorities and consumer organizations may receive structured referrals or verified evidence packages where the dispute cannot be resolved within the platform. The proposed implementation uses a permissioned distributed ledger rather than an unrestricted public blockchain. Validator nodes would be operated by authenticated institutions participating in the ODR ecosystem, such as the platform operator, accredited mediation bodies, consumer organizations or regulated service providers. This choice reduces unnecessary public disclosure, permits governance and access controls, and supports compliance with data-protection and procedural requirements. A low-energy Byzantine fault tolerant or crash fault tolerant consensus mechanism, such as a PBFT- or Raft-type protocol, is appropriate for a controlled network with known validators. The architecture does not depend on cryptocurrency mining or proof-of-work. The precise consensus mechanism should be selected during prototyping according to the number of validators, trust assumptions, fault tolerance and performance requirements. The AI services and case-management platform communicate through authenticated application programming interfaces. The blockchain connector converts approved procedural events into transactions and returns confirmation, status and integrity proofs to the off-chain platform. No AI model should be able to trigger irreversible smart-contract execution directly. Execution requires a validated instruction from the case-management layer and any required human authorisation. The proposal is technology-neutral at the conceptual level. A future prototype could use enterprise frameworks that support permissioned governance, channels, access control and auditable transaction histories. The article does not claim that a particular software stack has been implemented or benchmarked.

A central feature of the model is the functional separation between on-chain and off-chain processes:

- Off-chain domain: user interaction, AI processing, mediation, and data storage;
- On-chain domain: proof of dispute, validation, and enforcement.

This separation ensures compliance with data protection regulations; scalability and efficiency; preservation of blockchain’s integrity for critical functions only. The integration between layers is achieved through secure interfaces that translate off-chain outcomes into on-chain execution conditions.

**Table 2:** 

Component	Function	Typical data	Location	Primary safeguard	Human control
User interface	Complaint submission and guidance	User narrative, preferences, accessibility needs	Off-chain	Encryption and data minimisation	User may edit and withdraw before filing
AI assistance	Triage, classification, explanation	Claim text, evidence metadata, generated recommendations	Off-chain	Model governance, logging and confidence disclosure	Review for contested or high-impact outputs
ODR case management	Communication, negotiation and mediation	Pleadings, offers, notices, procedural record	Off-chain	Role-based access and retention limits	Mediator or legal reviewer
Evidence repository	Secure storage and integrity checking	Documents, communications, transaction exports	Off-chain, encrypted	Access controls, hashing and versioning	Disclosure subject to procedural rules
Dispute registry	Proof of filing and status	Hash, timestamp, pseudonymous case identifier	On-chain	No personal data; limited metadata	Correction through linked superseding record
Escrow contract	Temporary control of assets	Asset amount, state and release conditions	On-chain	Multi-signature and pause function	Release requires validated instruction
Execution contract	Implementation of settlement	Validated conditions and execution proof	On-chain	Waiting period and override protocol	Human authorisation where required
Audit layer	Traceability	Event logs, software versions, approvals	Hybrid	Immutable proofs plus detailed off-chain logs	Independent audit and supervisory access

Personal data, communications, evidence and AI-generated analyses should not be stored directly on-chain. They remain in an encrypted off-chain repository governed by retention, access and deletion rules. The ledger stores only the minimum information required to prove integrity, timing, escrow status or execution. A hash proves that a particular version of a document existed at a given time, but the original document remains necessary for interpretation and procedural use. If an off-chain record is corrected, the architecture should retain an auditable version history while respecting applicable rights and retention limits. Identity requirements should follow a risk-based model. Users may access general information anonymously, but filing a claim, accepting a settlement or authorizing asset transfer requires reliable authentication. Where available and legally appropriate, the system could interoperate with recognized electronic-identification services or digital identity wallets. Alternative verification pathways are necessary to avoid excluding users who cannot use a particular identity technology. Consent to AI assistance, data sharing, mediation and smart-contract execution should be separated. The user should receive a concise explanation of each function, the relevant risks and the consequences of withdrawal. Acceptance of platform terms must not be treated as blanket consent to automated decision-making or execution.

The architecture creates cybersecurity risks at the interface, API, AI, storage and smart-contract levels. Minimum controls include encryption in transit and at rest, multi-factor authentication for privileged roles, secure key management, code review, dependency monitoring, penetration testing, segregation of duties, rate limiting, anomaly detection and incident logging. Smart contracts should be independently audited before deployment and after material changes. Upgrade procedures must be transparent, and emergency pause functions must be protected by multi-party authorisation. The off-chain platform should maintain backups, recovery objectives and business-continuity procedures. An incident-response plan should specify detection, containment, preservation of evidence, user notification, regulatory reporting and restoration. Where an incident affects an active dispute, procedural deadlines and execution should be suspended until integrity is restored.

The platform should expose controlled, documented interfaces for exchanging case information with courts, regulators, mediators, insolvency administrators and CASPs. Interoperability concerns both technical format and legal authority. A technically valid data transfer is insufficient unless the receiving institution can lawfully use the information. The system should generate structured evidence packages containing the user’s statement, relevant transaction data, document hashes, procedural history and validation record. Export should be possible in human-readable and machine-readable formats. External authorities should not receive unrestricted access to the full platform; disclosure should follow role, purpose and legal basis. The architecture cannot guarantee enforcement against unidentified actors or assets outside technical or legal control. In such cases its value lies in early triage, evidence preservation, coordination and referral rather than automated recovery.

Not every crypto-asset dispute is suitable for the same ODR pathway. Before initiating the substantive dispute-resolution workflow, the platform should conduct an initial suitability assessment based on four minimum conditions: identifiability, jurisdictional pathway, evidentiary sufficiency, and technical and legal control over the contemplated outcome. Failure to satisfy one of these conditions should not necessarily terminate assistance; rather, it should determine which functions of the platform remain appropriate. Where the respondent is unidentified, the platform should shift from dispute resolution to evidence preservation and institutional referral. Where the relevant asset is outside the control of the platform, CASP or cooperating party, smart-contract enforcement should be disabled. Where jurisdiction is materially uncertain, the AI layer should communicate uncertainty rather than select a definitive forum autonomously. Where insolvency, criminal seizure or mandatory public-law proceedings apply, the system should refer the user to the competent court, administrator or authority. The architecture is therefore conceived as a bounded ODR infrastructure with explicit exit ramps, not as a universal mechanism for reversing blockchain transactions.

The architecture supports a structured dispute resolution workflow:

1. The user describes the event through an accessible interface and receives an explanation of the platform’s scope.
2. The AI layer identifies a provisional dispute category, missing information and potential urgency, while disclosing uncertainty.
3. The user confirms or corrects the classification and uploads or links relevant evidence.
4. The platform verifies evidence integrity and records a minimal timestamped proof on the ledger.
5. The case-management layer determines whether the matter is suitable for negotiation, mediation, referral or urgent escalation.
6. Parties negotiate with optional AI support and human mediation. Any automated suggestions remain non-binding.
7. A settlement is converted into a plain-language summary and, where appropriate, objectively verifiable execution conditions.
8. The parties confirm the terms. Required cooling-off, review and authorisation procedures are completed.
9. The smart contract releases, refunds or transfers controlled assets, or records the outcome for external enforcement.
10. The system provides a final record, explanation of execution and information on review or further remedies.

This workflow ensures continuity between user interaction, decision-making, and enforcement.

It embeds digital inclusion through specific design choices:

- epistemic accessibility: AI translates legal and technical complexity into understandable content;
- procedural guidance: users are supported at each stage of the process;
- cost reduction: automation minimizes reliance on expensive intermediaries;
- trust enhancement: transparent and automated enforcement increases confidence in outcomes;
- adaptability: the system accommodates varying levels of user expertise.

These features collectively reduce the structural barriers identified in Section 2.

While the architecture leverages automation, it does not eliminate the need for human judgment. Instead, it adopts a hybrid governance model in which AI supports analysis and interaction; smart contracts ensure execution; human actors retain authority over complex or contested decisions. This approach mitigates risks associated with algorithmic bias, rigidity, and lack of contextual sensitivity. At the same time, limitations remain, including dependence on data quality and system design; legal uncertainties surrounding smart contract enforceability; potential exclusion of users without basic digital access. Addressing these challenges requires ongoing refinement and regulatory alignment.

The proposed hybrid ODR model contributes to the literature and practice of digital justice by integrating AI and blockchain into a coherent dispute resolution framework; operationalizing digital inclusion as a design objective; bridging the gap between accessibility and enforceability; providing a scalable solution for cross-border crypto disputes. The architecture demonstrates how AI and smart contracts can be combined to create an inclusive, efficient, and trustworthy dispute resolution system tailored to the specific challenges of crypto-asset ecosystems. The following section applies this model to selected real-world scenarios to illustrate its practical implications.

## 6 Illustrative application scenarios and validation hypotheses of the hybrid ODR model

To illustrate the practical relevance and operational capacity of the proposed hybrid ODR architecture, this section considers three representative categories of crypto-asset disputes: (i) asset seizure and recovery, (ii) exchange failure and mass claims, and (iii) crypto scams and cross-border fraud. These case studies are illustrative rather than empirical. They are intended to show how the integration of AI, ODR mechanisms, and smart contracts could operate in different dispute contexts and how, in principle, these components may contribute to addressing specific forms of digital exclusion, improving procedural accessibility, and supporting more efficient and enforceable dispute-resolution processes. Accordingly, the scenarios do not establish the effectiveness of the proposed architecture in practice, but rather identify its potential functions, advantages, and limitations.

The following scenarios perform an analytical rather than evidentiary function. Each is used to generate a set of validation hypotheses for later empirical testing. The architecture is therefore not considered successful because it appears capable of processing the scenario conceptually. Success would require measurable improvement against an identified comparator under controlled or real-world testing.

### 6.1 Asset seizure and recovery

Cases involving the seizure of a substantial amount of cryptocurrency raise complex issues concerning asset tracing, evidentiary standards, legal accessibility, and the identification, freezing, and recovery of crypto-assets linked to illicit activity. Within the proposed hybrid ODR model, such disputes could be potentially be managed as follows:

- AI Assistance Layer: users could be supported in understanding asset tracing mechanisms, the legal basis for seizure, and available remedies. AI tools could assist in interpreting transaction histories and in providing plain-language explanations of complex blockchain data.
- ODR Layer: claims could be structured, and relevant evidence could be organized and assessed through a combination of automated tools and human oversight, facilitating interaction between parties, legal professionals, and competent authorities.
- Smart Contract Layer: where legally permissible and technically feasible, disputed assets could be placed in escrow, with smart contracts supporting conditional release on the basis of an agreed settlement, arbitral outcome, or judicial decision.

This configuration could reduce users’ dependence on highly specialized legal and technical expertise and may therefore contribute to improving access to justice for individuals who might otherwise encounter significant barriers to effective participation. Whether such benefits would materialize in practice, however, would depend on factors including the applicable legal framework, the availability and control of the relevant assets, the reliability of the evidence, and the accessibility of the technological interface.

Validation hypothesis: user-relative explanations and structured transaction visualization will improve comprehension of the legal and technical basis of asset restraint compared with conventional written information.

### 6.2 Exchange failure and mass claims

The collapse or insolvency of crypto-asset service providers represents a second category of disputes, often characterized by large numbers of affected users, fragmented claims, and complex insolvency or recovery procedures. In such cases, users may experience substantial difficulties in understanding their procedural position and recovering their funds. The proposed hybrid ODR architecture could offer several potential functions:

- AI Assistance Layer: it could provide standardized guidance for claim submission, helping users understand their rights, the available remedies and the procedural steps required to participate in recovery procedures.
- ODR Layer: it could support the aggregation, categorization, and management of large volumes of claims, potentially facilitating collective or coordinated dispute resolution and reducing certain administrative burdens.
- Smart Contract Layer: where legally appropriate, smart contracts could assist in the distribution of recovered assets according to predetermined and legally validated criteria, thereby potentially improving transparency and consistency in the allocation process.

By structuring and partially automating aspects of mass claims, the model could offer a means of addressing procedural complexity and managing large numbers of claims more systematically. This suggests potential advantages in terms of scalability and digital inclusion, particularly where conventional procedures are difficult for individual claimants to navigate. Nevertheless, these potential advantages would require empirical testing, particularly in relation to system capacity, user accessibility, procedural fairness, and compatibility with insolvency and consumer-protection rules.

Validation hypothesis: compared with a conventional electronic claims form, the hybrid workflow will reduce incomplete submissions and average claim-processing time without producing statistically significant deterioration in comprehension, fairness perception or error rates.

### 6.3 Crypto scams and cross-border fraud

Crypto scams, particularly those involving sophisticated transnational networks, constitute a third category of disputes with significant implications for digital inclusion and access to remedies. Victims of phishing attacks, investment fraud, fraudulent crypto schemes, and laundering operations involving multiple jurisdictions frequently face difficulties in identifying responsible actors, tracing assets, understanding jurisdictional rules, and accessing appropriate redress mechanisms. Within the proposed architecture, the different layers could potentially support victims in several ways:

- AI Assistance Layer: could assist in identifying patterns associated with fraudulent activity, highlighting potential scam indicators, and guiding users through preliminary steps for reporting the incident and preparing a claim.
- ODR Layer: could provide a structured environment for managing dispute-related information and communications, including in cross-border settings, and could facilitate coordination between affected parties and relevant institutional actors where such cooperation is legally and practically possible.
- Smart Contract Layer: where assets are identifiable, recoverable, and subject to lawful control, smart-contract mechanisms could support restitution processes, including escrow-based arrangements or conditional transfers.

Validation hypothesis: AI-supported evidence organization and triage will improve users’ ability to identify relevant evidence and appropriate escalation pathways compared with unguided reporting, without increasing false confidence in recoverability.

This integrated configuration could offer a more accessible and coordinated framework for responding to certain forms of cross-border crypto-related harm. Its usefulness would, however, be highly dependent on the availability of identifiable counterparties, jurisdictional cooperation, asset traceability, and the existence of legally enforceable recovery mechanisms. In situations involving anonymous perpetrators, inaccessible wallets, non-cooperative actors, or conflicting jurisdictions, the contribution of the architecture would necessarily be more limited.

Across the three case studies, several recurring features can be identified. AI-assisted guidance could potentially reduce informational asymmetries and help users navigate technically complex disputes. Structured ODR environments may improve procedural accessibility and organise interactions among parties and institutions. Smart-contract mechanisms may support the execution of certain legally validated outcomes where the relevant assets are technically and legally capable of automated management. The scenarios also suggest that the architecture could accommodate both individual and large-scale claims, although its actual scalability would need to be evaluated through technical testing and empirical deployment.

At the same time, the case studies underline the importance of hybrid governance. Automated processes cannot operate independently of human oversight, legal safeguards, institutional authority, and mechanisms for review and contestation. The illustrative application of the model therefore suggests that its potential value lies not in replacing existing legal institutions, but in providing a complementary digital infrastructure through which certain procedural, informational, and enforcement-related barriers may be reduced. Taken together, these scenarios illustrate how the proposed hybrid ODR model could be adapted to different categories of crypto-asset disputes and indicate its potential to contribute to a more accessible and user-centered dispute-resolution environment.

By integrating AI assistance, structured ODR processes, and smart-contract functionality within a coherent framework, the model offers a conceptual response to the technical, legal, and cognitive barriers identified in the preceding sections. The case studies should not, however, be interpreted as empirical confirmation of the model’s effectiveness, scalability, inclusiveness, or superiority over existing dispute-resolution mechanisms.

Its actual effectiveness would depend on implementation within appropriate legal and regulatory environments, institutional cooperation, technological reliability, user acceptance, and the continuous evaluation and refinement of its components. Future empirical research would therefore be required to assess, inter alia, usability, accessibility, procedural fairness, scalability, cost, decision quality, user trust, and enforceability in real or controlled dispute-resolution settings. Accordingly, the case studies support a more limited but methodologically consistent conclusion: the proposed architecture appears sufficiently adaptable at a conceptual level to be applied to different real-world dispute typologies and has the potential to support more scalable and inclusive forms of digital dispute resolution. Whether this potential can be realized in practice remains an empirical question.

The analysis of the three scenarios also suggests that the potential applicability of the architecture is contingent upon four key conditions: the dispute can be legally characterized with sufficient precision; relevant evidence can be identified and preserved; at least one responsible, identifiable, or cooperative actor can participate in the process; and the asset or dispute outcome is capable of lawful implementation. Its potential value decreases significantly where identity, jurisdiction, asset control, evidentiary reliability, or legal authority is absent. These limitations should be made transparent to users from the outset and incorporated into the design of the system through eligibility criteria, risk disclosures, and appropriate escalation to human or institutional decision-makers.

## 7 Legal and regulatory implications

The integration of AI and smart contracts into dispute resolution for crypto-asset ecosystems raises complex legal and regulatory questions. While the hybrid ODR architecture proposed in this paper offers significant potential to enhance digital inclusion, its deployment must be aligned with evolving legal frameworks governing artificial intelligence, crypto-assets, data protection, and access to justice. This section examines key regulatory dimensions and highlights the challenges associated with compliance, accountability, and legitimacy.

AI systems used in legal and dispute resolution contexts are increasingly subject to regulatory oversight, particularly within the European Union (EU). The regulatory classification of the AI layer depends on its intended purpose and deployment context rather than merely on its use within an ODR platform. AI used only for general legal information, administrative assistance, document organization or preliminary non-binding triage would not automatically constitute a high-risk system. By contrast, where an AI system is intended to support an ADR body in researching or interpreting facts and law and applying the law to a concrete dispute in a manner capable of producing legal effects, the high-risk classification under the EU AI Act may become relevant. The proposed architecture therefore separates informational and organizational assistance from legally consequential assessment. Even where an AI component falls outside the high-risk category, transparency, data-protection and consumer-law obligations may remain applicable.

High-risk classification entails a series of obligations, including transparency requirements (ensuring that users are aware they are interacting with AI systems), explainability and documentation (enabling users and regulators to understand system logic), risk management and monitoring (including ongoing assessment of system performance), and human oversight (ensuring that automated processes remain subject to meaningful control).

These requirements align with the design principles of the hybrid ODR architecture, particularly its emphasis on explainable AI and human-in-the-loop mechanisms. However, compliance imposes additional burdens in terms of system design, documentation, and auditing, which must be carefully integrated into the development process.

The legal environment for crypto-assets is undergoing rapid transformation, with the adoption of frameworks such as the Markets in Crypto-Assets (MiCA) Regulation in the European Union, establishing rules governing crypto-asset service providers (CASPs), including requirements related to transparency, consumer protection, and operational resilience (Szostek and Prabucki, 2025; Bygrave and Schmidt, 2025).

The proposed ODR platform may interact with crypto-asset service providers subject to requirements concerning governance, information, complaint handling, consumer protection and operational resilience. The platform is not necessarily itself a financial intermediary, but its legal status will depend on the services it provides and its control over assets. Integration with a regulated service provider may improve access to account information, complaint handling and execution. It may also create dependencies and conflicts of interest. Governance arrangements should preserve the procedural independence of mediators and ensure that the service provider cannot alter records or prevent valid escalation (de Caria et al., 2025). A central issue concerns the legal recognition and enforceability of smart contracts. While smart contracts can execute transactions on a blockchain, their status as legally binding agreements varies across jurisdictions. Key-questions include whether code-based agreements satisfy traditional requirements of contract law (e.g., consent, intention, legality); how errors, bugs, or unintended outcomes should be treated; how disputes involving smart contracts themselves can be resolved. In the hybrid ODR model, smart contracts are primarily used as enforcement tools rather than as autonomous legal instruments (Governatori et al., 2018; De Caria, 2018). This functional approach mitigates some legal uncertainties by linking smart contract execution to outcomes determined through human-mediated or legally recognized processes. Nevertheless, ensuring that smart contract outputs are legally valid and enforceable in off-chain contexts remains a critical challenge.

The allocation of liability within AI- and blockchain-based systems is a complex and evolving issue. In the proposed architecture, multiple actors may be involved, including developers of AI systems and smart contracts, operators of the ODR platform, users interacting with the system, external mediators or legal professionals. Determining responsibility in cases of malfunction, bias, or incorrect outcomes requires clear governance structures. For example, if an AI system provides misleading legal guidance, liability may arise from design or training deficiencies; if a smart contract executes an erroneous transaction, responsibility may depend on coding errors or user input; if human oversight fails, accountability mechanisms must address procedural deficiencies.

Emerging regulatory approaches emphasize the need for traceability, documentation, and auditability to support accountability. The hybrid model’s layered design, particularly its separation between off-chain and on-chain processes, can facilitate such mechanisms.

The processing of personal and transactional data within ODR systems raises significant data protection concerns. In jurisdictions such as the European Union, the General Data Protection Regulation (GDPR) imposes strict requirements regarding lawful processing and consent; data minimization and purpose limitation; rights of access, rectification, and erasure; restrictions on automated decision-making.

The immutable nature of blockchain records creates tensions with these principles, particularly regarding the right to erasure. This approach enhances compliance while preserving the benefits of blockchain-based verification (Pagallo et al., 2018).

Beyond technical compliance, the deployment of AI and smart contracts in dispute resolution must satisfy broader principles of procedural fairness and access to justice (Ortolani, 2019; Schmitz and Rule, 2019; Ast and Deffains, 2020). These include the right to be heard and to present evidence; the right to an impartial decision-maker; transparency of processes and outcomes; the possibility of review or appeal.

Automated systems risk undermining these principles if not carefully designed. The hybrid ODR model addresses this concern by integrating human oversight at critical stages; ensuring explainability of AI outputs; providing mechanisms for contestation and escalation.

In this way, the model seeks to align technological innovation with fundamental legal values.

Crypto-asset disputes frequently involve parties and assets located in multiple jurisdictions, raising issues of applicable law, jurisdiction, and enforcement. ODR systems have the potential to facilitate cross-border dispute resolution by providing standardized procedures and digital access points.

However, legal recognition of ODR outcomes varies across jurisdictions, enforcement of decisions may still require interaction with national legal systems and regulatory fragmentation can create uncertainty for users. The hybrid model can mitigate some of these challenges by combining globally accessible digital processes with locally compliant legal frameworks, but further harmonization is needed to fully realize its potential. Overall, the proposed hybrid ODR architecture aligns with emerging regulatory trends that emphasize responsible AI development, consumer protection in digital finance, and the integration of technological and legal infrastructures. However, its successful implementation requires continuous adaptation to evolving legal standards and active engagement with regulators, practitioners, and users. The legal and regulatory implications of integrating AI and smart contracts into dispute resolution are both enabling and constraining. While existing frameworks provide important guidance, they also highlight the need for careful system design, robust governance, and ongoing evaluation. The next section addresses this need by proposing a framework for assessing the effectiveness and inclusiveness of the hybrid ODR model.

## 8 Evaluation frameworks for inclusive hybrid ODR systems

A smart contract may execute a transaction, but legal enforceability depends on consent, capacity, legality, applicable mandatory rules and the relationship between code and the parties’ agreement (Governatori et al., 2018). For this reason, the proposed architecture uses smart contracts to implement validated outcomes rather than to define the legal merits of the dispute. The plain-language settlement should remain the authoritative statement of the parties’ agreement. The code should be generated or reviewed against that text, and discrepancies should be resolved in favor of the legally validated agreement. Execution logs should make the relationship between the text and the code auditable. The layered architecture involves multiple actors: the platform operator, AI provider, data supplier, smart-contract developer, validator, mediator, legal reviewer, CASP and user. Accountability may become diluted unless control is mapped explicitly. The platform should maintain a responsibility matrix that identifies who selects and updates models, approves smart-contract code, authorizes execution, responds to incidents and handles complaints. Logs should record the software version, input, output, reviewer and execution authorization associated with each relevant event. Liability will depend on applicable law and the circumstances of the failure. The architecture cannot predetermine that allocation, but it can preserve the traceability required to assess it.

The platform processes personal, financial and transactional data and must comply with principles of lawfulness, fairness, transparency, purpose limitation, minimization, accuracy, storage limitation, security and accountability. Restrictions on solely automated decisions may also be relevant where outputs produce legal or similarly significant effects. Blockchain immutability may conflict with rights of correction and erasure. The proposed architecture therefore keeps personal data and evidence off-chain and records only limited hashes, timestamps and execution proofs. A hash may still be personal data in some contexts, so access, linkage and retention require careful governance. Data subjects should receive clear information on the controller, purposes, recipients, retention periods, automated functions and available rights. Cross-border transfers and disclosures to external authorities require a valid legal basis. Digital dispute resolution must protect the right to be heard, equality of arms, impartiality, reasoned outcomes, access to evidence and the possibility of review. Efficiency does not justify eliminating these safeguards. The architecture supports procedural fairness through user-relative explanations, correction mechanisms, human escalation, waiting periods before execution, transparent settlement terms and export to formal proceedings. Vulnerability should trigger additional assistance rather than reduced procedural choice. The platform should also disclose its institutional relationships, funding and any economic incentives associated with settlement or transaction execution.

A digital platform may be globally accessible, but access does not resolve applicable law, jurisdiction or recognition. The system should avoid presenting a single legal route as universally valid. Triage should identify connecting factors and explain uncertainty. Outcomes may be implemented directly only where the relevant assets and parties are within technical and legal control. Other outcomes may require contractual enforcement, arbitration, court recognition, insolvency procedures or regulatory action. The system should preserve a clear boundary between platform settlement and public adjudication. Therefore, the architecture should be evaluated through technical, legal and user-centered measures. Because the current study is conceptual, the indicators below are proposed for future prototyping and do not represent measured results:

**Table 3:** 

Dimension	Illustrative indicators	Method	Relevant users	Indicative success question	Indicative benchmark
Accessibility	Completion across devices; language availability; assistive-technology compatibility; abandonment rate	Accessibility audit, task observation, log analysis	Users with disabilities, low digital literacy, mobile-only users	Can diverse users enter and complete the process?	WCAG 2.2 AA; ≥90% critical-task completion
Comprehension	Correct understanding of rights, options, AI limitations and settlement terms	Comprehension questions, interviews, think-aloud testing	Non-expert and vulnerable users	Do users understand enough to make an informed choice?	≥80% correct comprehension
Usability	Task completion time; errors; help requests; satisfaction	Usability study and validated scales	All participant groups	Is the process clear and manageable?	SUS ≥ 70
Fairness	Error rates across groups; challenge outcomes; differential escalation	Bias audit, case review, qualitative analysis	Language, age, disability and experience groups	Does the system treat comparable users consistently?	No materially unexplained group disparity
Contestability	Correction rate; review availability; reversal rate; response time	Procedural audit and user testing	Users disputing AI outputs	Can users effectively challenge and correct the system?	100% of legally significant AI outputs reviewable
Efficiency	Resolution time; procedural steps; administrative cost	Controlled simulation or comparative pilot	Users, mediators and operators	Does the model reduce avoidable time or cost?	Improvement over conventional workflow without reducing fairness metrics: statistically meaningful reduction compared with baseline without deterioration in fairness
Enforceability	Successful escrow and execution; failed transactions; post-outcome disputes	Sandbox test, smart-contract audit, case follow-up	Parties and platform operators	Are validated outcomes implemented reliably?	Near-complete execution of technically valid instructions; zero unauthorized irreversible execution
Security and resilience	Vulnerabilities; incident response time; recovery performance	Penetration testing, code audit, resilience exercise	Operators and institutional partners	Can the system resist and recover from attacks or failure?	No unresolved critical vulnerabilities before deployment
Trust and legitimacy	Perceived impartiality; willingness to use; understanding of governance	Survey, interview and focus group	Users and external stakeholders	Do participants regard the process as legitimate?	No deterioration relative to human-assisted comparator

The effectiveness of the proposed hybrid ODR architecture depends not only on its conceptual soundness but also on its performance in practice. To ensure that the system genuinely enhances digital inclusion, it is necessary to adopt a multidimensional evaluation framework capable of assessing both technical performance and legal-ethical outcomes. This section proposes a set of criteria and metrics designed to evaluate the architecture across five key dimensions: accessibility, usability, fairness, efficiency, and enforceability.

Accessibility refers to the extent to which users, in particular vulnerable and non-expert individuals, can access and engage with the system. This includes ease of entry into the platform; availability across devices and connectivity conditions; inclusiveness of design (e.g., language, disability support). Accessibility is a foundational condition for digital inclusion and must be assessed independently of other performance indicators.

Usability focuses on the user experience and the clarity of interaction with the system. In the context of AI-supported dispute resolution, this includes intelligibility of AI outputs; effectiveness of guided workflows; user comprehension of legal and procedural steps. Given the emphasis on explainability, usability must be closely linked to epistemic accessibility, measuring whether users can meaningfully understand and act upon the information provided.

Fairness concerns the extent to which the system produces equitable outcomes and avoids discriminatory effects. Evaluation should consider presence of algorithmic bias in classification or recommendations; consistency of outcomes across user groups; availability of mechanisms for contestation and review. This dimension is particularly important in ensuring that AI systems do not reproduce or exacerbate existing inequalities.

Efficiency measures the system’s ability to reduce the time and cost associated with dispute resolution. Relevant indicators include average time to resolution; reduction in procedural steps; cost savings compared to traditional legal processes. Efficiency is essential for scalability and for making dispute resolution accessible to users with limited resources.

Enforceability refers to the reliability of outcome execution, particularly through smart contract mechanisms. This includes success rate of automated enforcement; integrity and auditability of records; user trust in system outcomes. Trust is a critical factor in user adoption and is closely linked to transparency and reliability. To operationalize these dimensions, the following metrics can be employed:

- User comprehension rate: percentage of users who correctly understand their legal position after interacting with the system;
- Resolution time: average duration from complaint submission to outcome execution;
- Cost reduction index: comparison between ODR and traditional dispute resolution costs;
- Bias indicators: statistical measures of differential outcomes across user groups;
- User satisfaction scores: feedback on usability, fairness, and trust;
- Enforcement reliability rate: proportion of cases successfully executed via smart contracts.

These metrics provide a basis for both quantitative and qualitative evaluation, but the proposed framework can be implemented through a combination of methodologies:

- User testing and usability studies, focusing on interaction and comprehension;
- Experimental simulations, modeling dispute scenarios and system responses;
- Case-based evaluation, applying the system to real or realistic disputes;
- Auditing and monitoring, assessing algorithmic performance and compliance over time.

A mixed-methods approach is particularly valuable in capturing both technical performance and user experience (O'Brien and Lebow, 2013).

A future evaluation should combine usability testing, simulated disputes, legal expert review, security testing and participatory assessment. Initial testing could compare the hybrid interface with a conventional complaint form, while holding the dispute scenario constant. This would permit measurement of comprehension, completion, error and time differences. Participants should include non-expert consumers, users with limited digital literacy, persons with accessibility needs, SMEs, mediators, lawyers, CASP complaint personnel and regulators. Recruitment should avoid relying exclusively on technologically experienced crypto users. The evaluation should include pre-defined stopping rules for safety or fairness failures. Any pilot involving real assets should begin in a sandbox with nominal or simulated value. Independent ethics, data-protection and cybersecurity review should precede live deployment.

Evaluation does not end at deployment. The operator should monitor model drift, differential error, complaints, security incidents, failed executions, human-review rates and user abandonment. Material changes to models or smart contracts require renewed testing. Monitoring should also assess whether automation changes user behavior in unintended ways, including excessive deference to recommendations, reduced willingness to seek human advice or pressure to settle. The relevant question is not only whether the technology works, but whether it improves the user’s practical capacity to obtain a fair and enforceable remedy.

Given the evolving nature of AI and blockchain technologies, evaluation should be understood as an iterative process. Continuous monitoring and feedback loops are necessary to identify weaknesses, adapt system design, and ensure ongoing compliance with legal and ethical standards. Moreover, evaluation should incorporate participatory approaches, involving users, legal practitioners, and other stakeholders in the assessment process. This is particularly important for identifying forms of exclusion that may not be visible through purely technical metrics. Ultimately, the purpose of the evaluation framework is to determine whether the hybrid ODR model effectively promotes digital inclusion. This requires moving beyond technical performance to assess whether users can meaningfully access and understand the system; whether outcomes are fair and enforceable; whether the system reduces structural barriers to justice. In this sense, evaluation becomes a tool not only for system optimization but also for normative assessment, ensuring alignment with access to justice principles.

## 9 Limitations, conclusions and future researches

This study has several limitations. First, it is conceptual and design-oriented. The proposed architecture has not been implemented, and no claims are made regarding measured improvements in accessibility, resolution time, cost, fairness or enforcement. Second, the technical specification remains architecture-level rather than software-level. The choice of permissioned ledger, consensus mechanism, identity service, AI model and storage infrastructure must be validated through prototyping, threat modeling and performance testing. Third, the application scenarios are illustrative and do not constitute empirical case studies. They demonstrate possible workflows and limits but cannot establish effectiveness across jurisdictions or dispute populations. Fourth, the architecture depends on legal and technical control. It cannot identify every pseudonymous actor, reverse transactions outside controllable systems, recover dissipated assets or replace courts, regulators, insolvency administrators and law-enforcement authorities. Fifth, the regulatory analysis is necessarily general. The classification of the platform, its AI components and its smart-contract functions will depend on the final deployment, institutional relationships, applicable law and the nature of the assets or services involved.

Crypto-asset disputes expose a gap between technological participation and practical access to remedies. Users may be able to acquire and transfer digital assets while remaining unable to understand a loss, formulate a claim, preserve evidence, identify a forum or enforce an outcome. The hybrid ODR architecture proposed in this article addresses that gap by combining an off-chain AI assistance layer with a minimized on-chain verification and enforcement layer. AI supports information, triage, explanation, evidence organization and negotiation. Human reviewers retain authority over contested legal questions and irreversible steps. Smart contracts provide registration, escrow and execution only where the conditions are clear, validated and legally appropriate.

The architecture’s central contribution is access to remedies by design. Accessibility, comprehension, contestability, human oversight, evidentiary integrity and enforceability are treated as connected system requirements. A platform that explains rights but cannot implement outcomes is incomplete; a platform that executes outcomes without explanation and review risks reproducing exclusion. The proposal remains to be tested. Its value at this stage lies in making the normative and technical requirements explicit and therefore open to evaluation. AI and smart contracts should not be understood as substitutes for justice institutions. They can contribute to fairer crypto-asset dispute resolution only when embedded in legally governed, secure, user-centered and contestable procedures [50].

Future researches should focus on the empirical validation of the proposed architecture, including pilot implementations, user testing, and cross-jurisdictional analysis. As digital financial ecosystems continue to evolve, the development of inclusive, transparent, and enforceable dispute resolution mechanisms will be critical to ensuring trust, fairness, and sustainability in decentralized environments. Scholars should develop a minimum viable prototype, conduct participatory co-design with vulnerable and non-expert users, test the system in simulated disputes, compare it with conventional complaint procedures and assess interoperability with regulated service providers and public institutions. Regulatory sandboxes may provide an appropriate environment for evaluating bounded execution under supervision. Further comparative work should examine how contract law, consumer protection, civil procedure, arbitration, insolvency and data protection affect the same architecture in different jurisdictions. Research should also evaluate governance models capable of preserving independence where the ODR platform is funded or operated by industry participants.

## Data Availability

The raw data supporting the conclusions of this article will be made available by the authors, without undue reservation.
